# Assessing patterns of change in lifestyle behaviours by parity: a longitudinal cohort study

**DOI:** 10.1093/ije/dyac139

**Published:** 2022-07-01

**Authors:** Maureen Makama, Arul Earnest, Siew Lim, Helen Skouteris, Briony Hill, Helena Teede, Jacqueline A Boyle, Wendy J Brown, Allison M Hodge, Lisa J Moran

**Affiliations:** Monash Centre for Health Research and Implementation, Monash University, Clayton, Victoria, Australia; Department of Epidemiology and Preventive Medicine, Monash University, Melbourne, Victoria, Australia; Monash Centre for Health Research and Implementation, Monash University, Clayton, Victoria, Australia; Health and Social Care Unit, School of Public Health and Preventive Medicine, Monash University, Melbourne, Victoria, Australia; Warwick Business School, Warwick University, Coventry, UK; Health and Social Care Unit, School of Public Health and Preventive Medicine, Monash University, Melbourne, Victoria, Australia; Monash Centre for Health Research and Implementation, Monash University, Clayton, Victoria, Australia; Monash Centre for Health Research and Implementation, Monash University, Clayton, Victoria, Australia; School of Human Movement and Nutrition Sciences, University of Queensland, St Lucia, Queensland, Australia; Cancer Epidemiology Division, Cancer Council Victoria, Melbourne, Victoria, Australia; Centre for Epidemiology and Biostatistics, Melbourne School of Population and Global Health, The University of Melbourne, Parkville, Victoria, Australia; Monash Centre for Health Research and Implementation, Monash University, Clayton, Victoria, Australia

**Keywords:** Parity, weight, diet quality, energy intake, physical activity, sitting time, longitudinal cohort study

## Abstract

**Background:**

The time constraints and reprioritization of personal health associated with having children may lead women to adopt less healthy lifestyles. We assessed the patterns of change in weight and lifestyle behaviours associated with having children and whether these differ between primiparous and multiparous women.

**Methods:**

Data were from Surveys 3 and 5 of the 1973–1978 birth cohort of the Australian Longitudinal Study on Women’s Health. In women who were nulliparous at Survey 3, we assessed changes in weight, energy intake, diet (diet quality, macronutrients and micronutrients), physical activity and sitting time by parity status at Survey 5 using one-way analysis of covariance.

**Results:**

Of 4927 eligible women, 2503 gave birth (1090 primiparous and 1413 multiparous) by Survey 5. Women who had given birth 6 years later increased weight (1.0 kg; 95% CI 0.5, 1.5), energy intake (833.9 kJ/day; 95% CI 706.7, 961.1) and diet quality (1.5 units; 95% CI 0.8, 2.1), but decreased physical activity [–405.0 Metabolic Equivalent of Task.min/week; 95% CI –464.2, –345.8] and sitting time (–1.8 h/day; 95% CI –1.9, –1.6) (adjusted mean differences) relative to those who remained nulliparous. In subgroup analysis involving further stratification by parity, the increase in diet quality was only seen in women who became primiparous and the decrease in sitting time was more marked in multiparous women.

**Conclusion:**

Childbearing is associated with increased weight and energy intake, decreased physical activity, increased diet quality and decreased sitting time. More research targeting weight, energy intake and physical activity for improvement in women during the childbearing years is warranted.

Key Messages

- Women may adopt less healthy lifestyles after having children due to the demands of caring for infants and young children; however, there is a paucity of research on the patterns of these changes by parity status.
- We used data from the 1973–1978 birth cohort of the Australian Longitudinal Study of Women’s Health to assess the patterns of change in lifestyle behaviours by parity status.
- On average, women who became parous increased weight by 1.0 kg, energy intake by 833.9 kJ/day and diet quality by 1.5 units, but decreased physical activity by 405 Metabolic Equivalent of Task.min/week and sitting time by 1.8 h/day more than those who remained nulliparous over a 6-year period.
- Diet quality improved only in women who became primiparous whereas sitting time decreased progressively with higher parity.
- Energy intake increases and physical activity decreases in women during their childbearing years and should be targeted in interventions for weight management.

## Introduction

Obesity is a growing challenge to public health globally, and together with overweight, affects more than a third of the world’s population.^1^ The global prevalence of obesity in women increased from 6.4% to 14.9% between 1975 and 2014.^1^ In Australia, the prevalence of overweight and obesity in women aged ≥18 years was 59.7% in 2018^2^ with 47% of women who gave birth that year living with overweight or obesity.^3^ This is of concern as maternal obesity increases the risk of pregnancy complications, e.g. pre-eclampsia, gestational diabetes and later development of chronic diseases such as diabetes and cardiovascular disease in both mother and offspring.^4–6^ Previous longitudinal studies have reported greater weight gain in women of childbearing age than in older women.^7^^,^^8^ Several studies suggest childbearing contributes to weight gain in women due to excessive gestational weight gain and post-partum weight retention.^9^^,^^10^ Changes in lifestyle behaviours after having children may also play a role in this.^11^

Modifiable lifestyle behaviours including diet, physical activity and sedentary behaviours play a significant role in weight management and prevention of obesity^12^ with some of these worsening in women following birth of their children.^13^ Longitudinal studies exploring the magnitude of lifestyle change attributable to parity are scarce and have not explored energy intake, diet quality and sitting time^13–17^ or explored the effect of increasing parity. We therefore aimed to explore the prospective patterns of change in weight, diet quality, diet (diet quality, macronutrient and micronutrient intake), physical activity and sitting time associated with becoming parous and whether these differ between primiparous and multiparous women. Understanding how these lifestyle behaviours change with parity is necessary for better tailoring of lifestyle interventions for weight management to the specific needs of each parity group.

## Methods

### Study population

The Australian Longitudinal Study on Women’s Health (ALSWH) is a prospective cohort study of women’s physical and mental health and use of health services.^18^ In 1996, three birth cohorts of women, young (1973–1978), mid-age (1946–1951) and old (1921–1926) were randomly recruited from the national health insurance (Medicare) database that includes all Australian citizens and permanent residents. The women completed mailed surveys every 3 years. A fourth birth cohort (1989–1995; added in 2013) completed online surveys. Further details of the methods used and participants’ characteristics are provided elsewhere.^19^^,^^20^ For this analysis, we used data from the 1973–1978 birth cohort that began with 14 247 respondents. We included data from Surveys 3 (2003; aged 25–30 years) with 36.3% attrition and 5 (2009; aged 31–36 years) with 42.4% attrition since cohort establishment, as these focused on women of childbearing age. Survey 4 did not include the dietary questionnaire and was therefore not included.

### Parity

**Figure 1:**
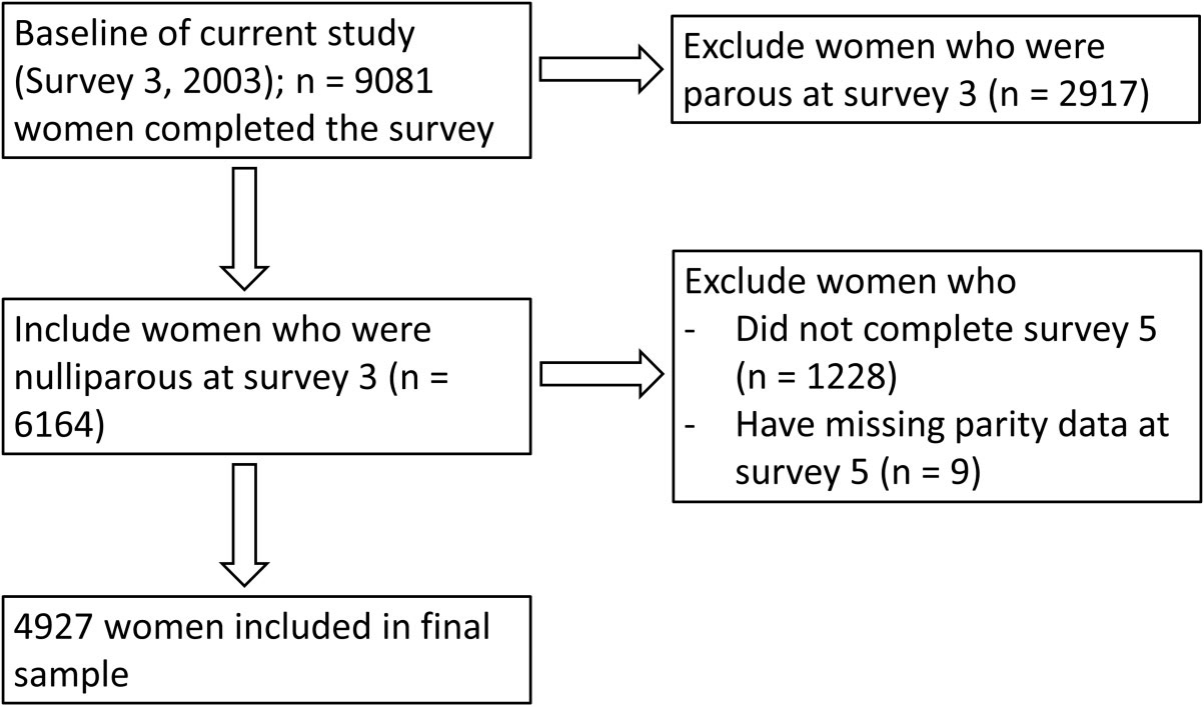
Flow chart of participant selection for analysis

Parity status at both surveys was self-reported. Only data from women who were nulliparous and not pregnant at Survey 3 were included in this analysis (Figure 1). Of 6164 eligible women, 1228 did not complete Survey 5 and 9 had missing data on parity status at Survey 5 and were therefore excluded from the analysis, leaving 4927 women in the final sample. Parity status was defined by number of children; pregnancy status was not considered in the definition. Women who gave birth between Surveys 3 and 5 were categorized as parous (*n* = 2503) [further divided into those who had only one child (primiparous, *n* = 1090) and those with more than one child (multiparous, *n* = 1413)] and women who remained childless as nulliparous (*n* = 2424).

### Outcome measures

The primary outcomes were changes in weight, energy intake, diet quality, physical activity and sitting time in women who became parous relative to those who remained nulliparous by Survey 5. The secondary outcomes were changes in macronutrient and micronutrient intake. Participants were asked to record their weight in kilograms without shoes and wearing light clothing. Dietary intake was assessed using the Dietary Questionnaire for Epidemiological Studies Version 2, a validated semi-quantitative food frequency questionnaire (FFQ). It used a 10-point response grid for participants to report usual frequency of consumption of 74 foods and 6 alcoholic beverages over the past 12 months. Serving sizes were adjusted according to portion-size photographs. Respondents were asked further questions about total number of daily serves of fruit, vegetables, bread, dairy products, eggs, fat spreads and sugars. Respondents (*n* = 286) with implausible energy intake defined as daily energy intake <2100 or >14 700 kJ/day were excluded from the analysis. Diet quality was measured by the Dietary Guideline Index (DGI) that reflects adherence to the Dietary Guidelines for Australian Adults.^21^ The DGI consists of 15 items including dietary indicators of vegetables and legumes, fruit, total cereals, meat and alternatives, total dairy, beverages, salt, saturated fat, alcoholic beverages and added sugars. Because the FFQ did not provide specific information to assess salt and fluid intake, these components were excluded. Also, the FFQ did not provide specific information on trimmings of fat from meat; therefore, DGI saturated fat was computed as (DGI lean meat + DGI dairy)/2. Each component was scored from 0 to 10, with 10 indicating an optimal intake (Supplementary Table S1, available as Supplementary data at *IJE* online). The total score was the sum of 13 indicators with the DGI having a possible range of 0 to 130. A higher score indicates better compliance with dietary guidelines.^21^

Physical activity was measured using items from Active Australia’s 1999 National Physical Activity Survey,^22^^,^^23^ which asks participants to report the frequency and duration of a variety of activities performed in the last week for ≥10 min. These included walking for transport or recreation, moderate and vigorous intensity leisure-time activity. Physical activity was calculated as the sum of the products of total weekly minutes in each of the three categories of physical activity and the metabolic equivalent value (MET.min/week) assigned to each category: [3.33 Metabolic Equivalent of Task (MET) × walking minutes] + (3.33 MET × moderate intensity activities minutes) + (6.66 MET × vigorous intensity activities minutes). Physical activity values were truncated at 5600 MET.min/week (equivalent to 1680 min/week). Participants were asked to report the time (hours and minutes) typically spent sitting down while doing things like visiting friends, driving, reading, watching television or working at a desk or computer on a usual weekday and separately on a usual weekend day. An estimate of the average daily sitting time was calculated as (weekday sitting × 5 + weekend sitting × 2)/7.

### Socio-demographic, health and behavioural variables

Self-reported measures included age (years); weight (kg); height (cm); marital status (categorized: married/de facto, separated/divorced/widowed or never married); smoking status [categorized: current smoker or current non-smoker (includes ex-smoker)]; annual household income [categorized: no, low (1–36 399), medium (36 400–77 999) or high income (>77 999) Australian dollars per annum]; occupation (categorized: no paid job, clerical/trade, associate professional or professional); education (categorized: no formal/high school, trade/diploma or degree and higher); alcohol intake [categorized based on risk of harm in the longer term associated with alcohol consumption using National Health and Medical Research Council guidelines as non-drinker, rarely drinks (less than monthly), low-risk drinker (≤14 drinks/week) or risky/high-risk drinker (≥15 drinks/week)]; country of birth (categorized: Australian-born or non-Australian-born). Geographic location was assessed using the Accessibility/Remoteness Index of Australia (ARIA+) (categorized: major cities, inner regional, outer regional, remote or very remote Australia). Depression was assessed using the 10-item Centre for Epidemiological Studies Depression Scale with a score of ≥10 used as a categorical cut-off for clinically significant symptoms.^24^ Anxiety was assessed using the anxiety subscale of the Goldberg Depression and Anxiety Scale with a score of ≥5 used as a categorical cut-off for risk of clinical anxiety. Stress was assessed using the Perceived Stress Questionnaire that has been previously validated in the ALSWH. Values were analysed as the mean of the multi-item summed score for perceived stress ranging from 0 to 4, with higher values referring to more stress.^25^

### Statistical analysis

Continuous variables were summarized as means with standard deviation whereas categorical variables were reported as percentages. Comparisons between women who became parous and those who remained nulliparous by Survey 5 were performed using independent *t*-tests for continuous and Chi-squared tests for categorical variables. Paired *t*-tests were used to assess changes in the outcome measures from Surveys 3–5 within nulliparous and parous groups and one-way analysis of covariance (ANCOVA) to assess differences between groups over time. The ANCOVA model was constructed by including covariates selected a priori based on known or suspected clinically relevant effects on energy intake, diet quality, physical activity or sitting time in existing literature and/or having *P*-values of ≤0.2 in univariable analysis. Variance inflation factor was used to assess multicollinearity. Household income was excluded from the model because it had a variance inflation factor of >10. The covariates included were age, body mass index (BMI), marital status, education, occupation, smoking status, alcohol intake, mean stress, depression, anxiety and the outcomes at baseline. ARIA+ was included as a covariate in diet-quality models. BMI was not included in the model for weight and alcohol intake was not included in the model where it was the outcome. In subgroup analysis, parity status at Survey 5 was further split into nulliparous, primiparous and multiparous. ANCOVA models and Tukey’s post hoc test were used to explore the differences in adjusted means (aMD) of the outcome measures across these groups.

We also conducted some sensitivity analyses. First, to capture uncontrolled confounding by pregnancy at Survey 5, women who were pregnant at Survey 5 (*n *=* *573) were excluded from analyses. Second, the DGI saturated fat component was excluded from the calculation of the diet-quality score in another sensitivity analysis. Third, using multiple imputation by chained equations, we repeated the ANCOVA analyses with imputed weight and BMI data to compare results with complete case analysis. Twenty imputations were performed. All *P*-values were calculated from two-tailed tests with a type 1 error rate of 5%. Analyses were performed using Stata software version 16 (Stata Corp, College Station, Texas, USA).

## Results

### Participant characteristics

Of the 4927 women who were nulliparous at Survey 3, 2503 became parous by Survey 5 whereas 2424 remained nulliparous. Participant characteristics by parity status at Survey 5 are reported in Table 1 and Supplementary Table S2 (available as Supplementary data at *IJE* online). At baseline (Survey 3), women who became parous by Survey 5 were slightly older, had lower BMI, were more likely to be married or in a de facto relationship, low-risk drinkers, medium-income earners, in professional jobs, have lower stress levels and were less likely to be smokers and have depression or anxiety than those who remained nulliparous.

**Table 1:** Participant characteristics at baseline (Survey 3) grouped by parity status at Survey 5

	Nulliparous at Survey 5 (*n *=* *2424)	Missing *n* (%)	Parous at Survey 5 (*n *=* *2503)	Missing *n* (%)	*P*-value
Age (years) [mean (SD)]	27.3 (1.4)	–	27.5 (1.4)	–	<0.001*
Weight (kg) [mean (SD)]	67.7 (16.2)	50 (2.1)	65.7 (12.8)	298 (11.9)	<0.001*
Height (cm) [mean (SD)]	165.7 (7.1)	3 (0.12)	166.1 (7.0)	7 (0.28)	0.031
BMI (kg/m^2^) [mean (SD)]	24.7 (5.7)	52 (2.1)	23.8 (4.4)	302 (12.1)	<0.001*
Country of birth		–		–	0.085
Australian-born	2225 (91.8)		2330 (93.1)		
Non-Australian-born	199 (8.2)		173 (6.9)		
Marital status [*n* (%)]		11 (0.45)		6 (0.24)	<0.001
Married/de facto	731 (30.3)		1830 (73.3)		
Separated/divorced/widowed	53 (2.2)		54 (2.2)		
Never married	1629 (67.5)		613 (24.6)		
Current smoker [*n* (%)]		10 (0.41)		7 (0.28)	<0.001
No	1797 (74.4)		2020 (80.9)		
Yes	617 (25.6)		476 (19.1)		
Alcohol intake [*n* (%)]		10 (0.41)		8 (0.32)	0.011
Low-risk drinker	1648 (68.3)		1763 (70.7)		
Non-drinker	149 (6.2)		132 (5.3)		
Rarely drinks	496 (20.6)		517 (20.7)		
Risky/high-risk drinker	121 (5.0)		83 (3.3)		
Education *n* (%)		26 (1.1)		51 (2.0)	0.362
No formal/high school	475 (19.8)		455 (18.6)		
Trade/diploma	546 (22.8)		593 (24.2)		
Degree or higher	1377 (57.4)		1404 (57.3)		
Annual household income *n* (%)		775 (32.0)		352 (14.1)	<0.001
No income	4 (0.2)		9 (0.4)		
Low (AUD 0–36 399)	296 (18.0)		213 (9.9)		
Medium (AUD 36 400–77 999	714 (43.3)		832 (38.7)		
High (AUD > 77 999)	635 (38.5)		1097 (51.0)		
Occupation [*n* (%)]		29 (1.2)		17 (0.7)	<0.001
No paid job	174 (7.3)		105 (4.2)		
Clerical job	473 (19.8)		442 (17.8)		
Associate professional	639 (26.7)		643 (25.9)		
Professional	1109 (46.3)		1296 (52.1)		
Stress level [mean (SD)]	1.0 (0.6)	4 (0.2)	0.8 (0.5)	4 (0.2)	<0.001*
Depression [*n* (%)]		30 (1.2)		25 (1.4)	<0.001
No	1732 (72.4)		1999 (81.0)		
Yes	662 (27.7)		469 (19.0)		
Anxiety [*n* (%)]		12 (0.5)		9 (0.4)	0.003
No	1082 (44.9)		1224 (49.1)		
Yes	1330 (55.1)		1270 (50.9)		
ARIA+^a^		9 (0.4)		4 (0.2)	0.109
Major cities of Australia	1530 (63.4)		1520 (60.7)		
Inner regional Australia	554 (22.9)		608 (24.3)		
Outer regional Australia	286 (11.8)		300 (12.0)		
Remote Australia	35 (1.5)		58 (2.3)		
Very remote Australia	10 (0.4)		13 (0.5)		

Data were analysed by *t*-test* for continuous variables and Chi-square or Fisher’s exact test as appropriate for categorical variables;

a ARIA+, Accessibility/Remoteness Index of Australia; SD, standard deviation; BMI, body mass index.

### Differences in primary outcomes over time by parity

Differences in the primary outcomes in women who became parous relative to those who remained nulliparous after 6 years are presented in Table 2. Although there was an increase in mean weight over 6 years, women who became parous gained more weight (aMD = 1.0 kg; 95% CI 0.5, 1.5). Energy intake decreased over time in women who remained nulliparous, but increased in those who became parous [aMD = 833.9 kJ/day (95% CI 706.7, 961.1) higher in parous than nulliparous women]. We observed a greater increase in diet quality over time for women who became parous than those who remained nulliparous (aMD = 1.5 units; 95% CI 0.8, 2.1). Physical activity decreased in both groups but more so in women who became parous (aMD = –405.0 MET.min/week; 95% CI –464.2, –345.8). Sitting time increased in women who remained nulliparous but decreased in those who became parous (aMD = –1.8 h/day; 95% CI –1.9, –1.6).

**Table 2:** Differences in weight, energy intake, diet quality, physical activity and sitting time over time grouped by parity status at Survey 5

Parameter	Survey 3 mean (SD)	Survey 5 mean (SD)	^a^Mean difference over time (95% CI)	*P*-value
**Weight** (kg)				
Nulliparous at Survey 5	67.7 (16.1)	71.3 (17.9)	3.6 (3.2, 3.9)	<0.001
Parous at Survey 5	65.7 (12.8)	69.6 (15.0)	3.9 (3.6, 4.2)	<0.001
^b^Unadjusted difference (Parous—Nulliparous) (*n* = 4550)			0.3 (–0.2, 0.8)	0.210
^c^Adjusted difference (Parous—Nulliparous) (*n* = 4351)			1.0 (0.5, 1.5)	<0.001
**Energy intake** (kJ/day)				
Nulliparous at Survey 5	6839.9 (2239.2)	6640.9 (2140.7)	–199.0 (–292.5, –105.5)	<0.001
Parous at Survey 5	6821.3 (2166.9)	7368.8 (2185.4)	547.5 (455.4, 639.7)	<0.001
^b^Unadjusted difference (Parous—Nulliparous) (*n* = 4792)			735.9 (625.6, 846.2)	<0.001
^c^Adjusted difference (Parous—Nulliparous) (*n* = 4263)			833.9 (706.7, 961.2)	<0.001
^d^ **Diet quality** (units)				
Nulliparous at Survey 5	83.9 (11.9)	87.4 (11.0)	3.4 (2.9, 3.9)	<0.001
Parous at Survey 5	84.9 (11.2)	88.7 (10.6)	3.8 (3.3, 4.3)	<0.001
^b^Unadjusted difference (Parous—Nulliparous) (*n* = 4793)			1.0 (0.4, 1.5)	<0.001
^c^Adjusted difference (Parous—Nulliparous) (*n* = 4253)			1.5 (0.8, 2.1)	<0.001
**Physical activity** (MET.min/week)				
Nulliparous at Survey 5	1198.8 (1153.3)	1037.6 (1066.5)	–161.2 (–214.3, –108.0)	<0.001
Parous at Survey 5	1077.5 (1066.1)	669.4 (790.8)	–408.1 (–454.8, –361.4)	<0.001
^b^Unadjusted difference (Parous—Nulliparous) (*n* = 4735)			–340.0 (–391.4, –288.7)	<0.001
^c^Adjusted difference (Parous—Nulliparous) (*n* = 4222)			–405.0 (–464.2, –345.8)	<0.001
**Sitting time** (h/day)				
Nulliparous at Survey 5	6.9 (2.8)	7.1 (2.8)	0.15 (0.03, 0.27)	0.0153
Parous at Survey 5	6.5 (2.7)	5.0 (2.5)	–1.5 (–1.6, –1.4)	<0.001
^b^Unadjusted difference (Parous—Nulliparous) (*n* = 4436)			–1.9 (–2.0, –1.8)	<0.001
^c^Adjusted difference (Parous—Nulliparous) (*n* = 3959)			–1.8 (–1.9, –1.6)	<0.001

a Data analysed using paired *t*-test for the within-group change; analysis of covariance was used to quantify the changes over time between nulliparous and parous women.

b Adjusted for the outcome measures at baseline.

c Additionally adjusted for age, BMI, marital status, education level, occupation category, smoking status, alcohol intake, mean stress level, depression and anxiety at baseline. BMI was not included in the model where weight was the outcome.

d Diet quality additionally adjusted for index of accessibility/remoteness. SD, standard deviation; BMI, body mass index; MET, Metabolic Equivalent of Task.

### Differences in secondary outcomes over time by parity

Over 6 years, the proportion of energy from carbohydrates decreased in both groups with a lesser decrease in women who became parous. Proportions of energy from fat, saturated fat, monounsaturated fat and cholesterol increased in both groups, with a greater increase in women who became parous. Fibre intake increased in women who became parous but did not change in those who remained nulliparous. In contrast, alcohol intake decreased in women who became parous but did not change in those who remained nulliparous. Glycaemic index decreased in both groups whereas glycaemic load decreased in women who remained nulliparous but increased in those who became parous (Supplementary Table S3, available as Supplementary data at *IJE* online). Iron intake increased in both groups, with a greater increase in women who became parous. Calcium intake increased in women who became parous but did not change in those who remained nulliparous. Folate and sodium intake decreased in women who remained nulliparous but increased in those who became parous (Supplementary Table S4, available as Supplementary data at *IJE* online).

### Differences in diet-quality components

Changes in diet quality varied across the DGI components. In both groups of women, there were improvements in the food variety, vegetables, whole grains, protein, dairy and saturated fat components. Among women who remained nulliparous, the lean protein and discretionary foods components improved; the cereals component worsened; and the low-fat dairy, alcohol, fruit and added sugars components remained unchanged. In contrast, among women who became parous, the cereals, alcohol and fruit components improved; the lean protein, low-fat dairy and added sugars components worsened; and the discretionary foods component remained unchanged (Supplementary Table S5, available as Supplementary data at *IJE* online).

### Differences by parity groups—nulliparous, primiparous and multiparous

**Figure 2:**
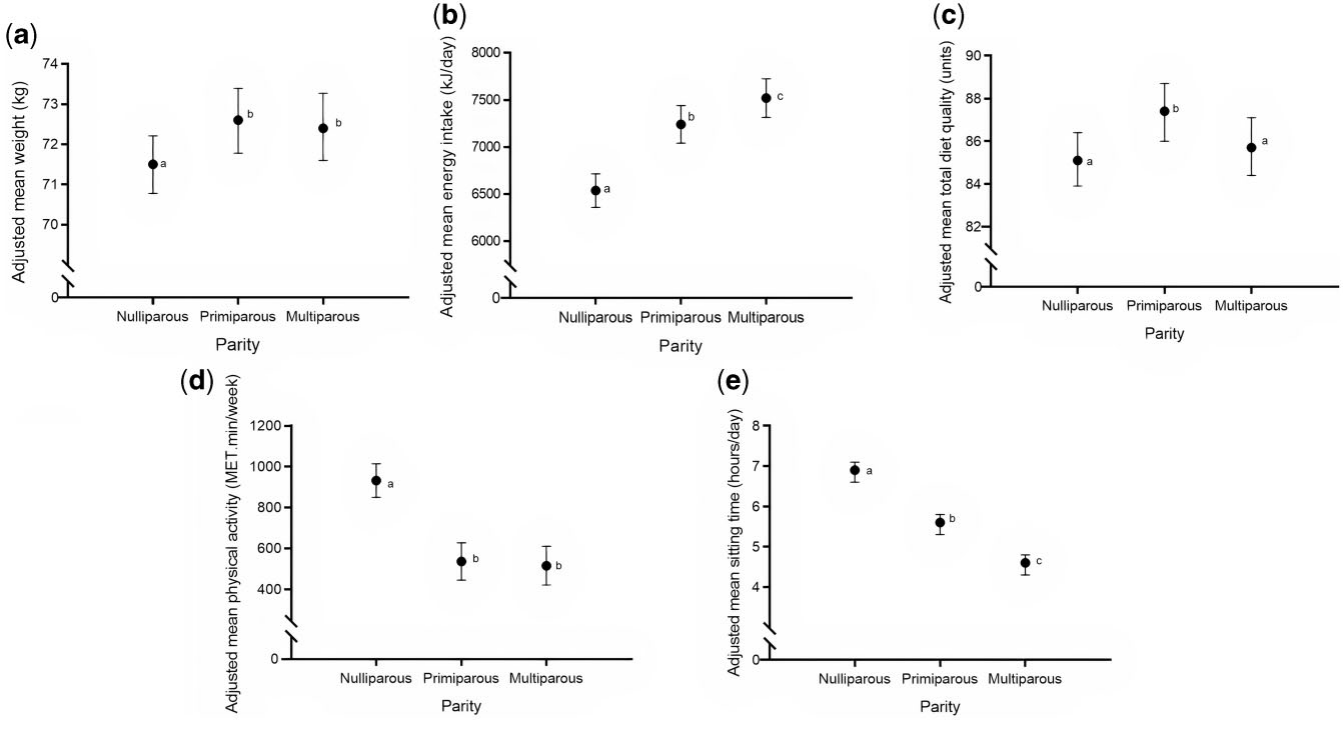
Adjusted mean (a) weight (kg), (b) energy intake (kJ/day), (c) total diet quality (units), (d) physical activity (Metabolic Equivalent of Task.min/week) and (e) sitting time (h/day) by parity status at Survey 5. Data are reported as marginal means and 95% confidence intervals of the outcomes estimated by parity groups—nulliparous, primiparous and multiparous—and were analysed by analysis of covariance adjusted for age, body mass index, marital status, education, occupation, smoking status, alcohol intake, mean stress level, depression, anxiety and outcomes at baseline (Survey 3). Diet quality was additionally adjusted for index of accessibility/remoteness. Differences in the characters indicate differences in the parity groups at *P* < 0.05 using Tukey’s post hoc comparison test. Refer to Supplementary Table S6 (available as Supplementary data at *IJE* online) for the numerical data.

Adjusted mean weight, energy intake, total diet quality, physical activity and sitting time by parity groups (nulliparous, primiparous and multiparous) are shown in Figure 2 and Supplementary Table S6 (available as Supplementary data at *IJE* online). Weight and energy intake were higher and physical activity lower in women who became primiparous or multiparous than in those who remained nulliparous (Figure 2a–d). Diet quality was higher in women who became primiparous, but not in those who became multiparous, than in those who remained nulliparous (Figure 2c and Supplementary Table S7, available as Supplementary data at *IJE* online). Sitting time was lower in women who became multiparous than in those who became primiparous and lower in those who became primiparous than in those who remained nulliparous (Figure 2e).

### Sensitivity analysis

In sensitivity analyses, we found no change in the magnitude or direction of effect sizes except for diet quality, which indicates confounding of associations of diet quality by pregnancy. After excluding pregnant women, the increase in diet quality was larger in women who became parous than in those who remained nulliparous (aMD = 1.7 units; 95% CI 1.0, 2.4) (Supplementary Table S8, available as Supplementary data at *IJE* online). The effect sizes based on imputed data were similar in direction and magnitude to that of complete case analyses (Supplementary Table S9, available as Supplementary data at *IJE* online).

## Discussion

In this longitudinal study of data from a population-based cohort of Australian women, we assessed changes in weight, energy intake, diet quality, physical activity and sitting time over time in relation to parity. For women who gave birth, we observed increases in weight and energy intake and decreases in physical activity, but improvements in diet quality and sitting time, relative to those who remained nulliparous. The improvements in diet quality were only observed in primiparous women and improvements in sitting time were greatest in multiparous women.

In our study, women who became parous gained 1.0 kg more than those who remained nulliparous after 6 years. Consistently with this, a recent longitudinal study reported greater weight gain in parous than nulliparous women ranging from 1.08 kg in primiparous women to 2.81 kg in women with at least four children.^16^ Earlier work by the ALSWH researchers reported that having a baby had a marked effect on 10-year weight gain (from 1996 to 2006), although part of this effect was attributable to changes made when the women married or started living with a partner.^9^

Energy intake that exceeds energy expenditure is a main driver of weight gain.^26^ Women who became parous increased energy intake by 833.9 kJ/day more than those who remained nulliparous with this difference contributing to the observed greater increases in weight for women who became parous relative to those who remained nulliparous. Consistently with this, Berge *et al.* reported a higher energy intake in women with children who were ≤5 years old than in nulliparous women (9874.2 vs 8834.5 kJ/day).^13^ We also report increases in proportions of energy from carbohydrates, fat, saturated fat and monounsaturated fat, but not protein and polyunsaturated fat, in women who became parous relative to those who remained nulliparous. Consistently with this, higher saturated fat intake (10.3% vs 9.5% total calories)^13^ was reported by women with children than by those without children, and exceeding recommended saturated fat intakes was associated with >3-fold higher odds of retaining ≥5 kg post-partum.^27^ Dietary protein may also aid weight management through increasing satiety and energy expenditure,^28^ with a 1% increase in protein and corresponding decreases in fats and carbohydrate resulting in decreases in energy intake of 213 and 134 kJ, respectively, in the general US population.^29^ It is therefore important to consider changes in dietary composition in addition to energy intake with regard to weight management. Women who became parous increased their iron, folate and calcium intakes and decreased alcohol intake, consistently with pregnancy dietary recommendations.

We report here that total diet quality improved by 1.5 units more in women who became parous than in those who remained nulliparous. This occurred through improvements in specific DGI components (increases in food variety, vegetables, fruit, cereal, protein and dairy, and decreases in saturated fat and alcohol) and worsening in others (decreases in whole grains, lean protein and low-fat dairy, and increases in added sugars and discretionary foods). As breastfeeding is a positive predictor of diet quality, these improvements may be partly attributable to dietary modifications to meet breastfeeding needs of the infant.^30^^,^^31^ The mean difference in total diet quality is very small and in subgroup analysis was only seen in women who became primiparous (aMD = 2.2 units; 95% CI 1.5, 3.0) (Supplementary Table S7, available as Supplementary data at *IJE* online). A previous study reported that a 10-unit increase in the DGI score was associated with lower systolic blood pressure (0.53 mmHg) and fasting glucose (0.003 mmol/L).^32^ This suggests that the observed 1.5-unit difference in diet quality (assessed as DGI) in this study is likely to be of minimal clinical relevance. The improvement in the DGI saturated fat component for women who became parous was not consistent with the reported increase in the proportion of energy from saturated fat in this study. This may be due to the absence of information on fat trimmings from meat in the calculation of DGI saturated fat. In sensitivity analysis excluding the DGI saturated fat component from the total diet-quality score, the effect size of diet quality was smaller (aMD = 1.3 units; 95% CI 0.7, 1.9). Although there is no prior research assessing diet quality and parity, a previous study reported that primiparous pregnant women received more dietary information and made more dietary changes than multiparous pregnant women.^33^ Our observation of an increase in weight despite improvements in diet quality for women who became primiparous is in agreement with some^31^^,^^34^ but not all^35^ studies reporting diet quality and weight change in post-partum women. However, as improvements in diet quality in women who became parous in our study were accompanied by increase in energy intake, this could negate any beneficial effect of diet quality on weight.

Physical activity is also a key driver of weight gain.^36^ On average, physical activity decreased by 405 MET.min/week more in women who became parous than in those who remained nulliparous after 6 years. This is consistent with previous studies that report an inverse association between physical activity and parenthood,^13^^,^^37–39^ and declines in physical activity during pregnancy that are sustained at lower levels post-partum.^12^^,^^40^^,^^41^ Women with young children face unique barriers to engaging in physical activity such as time constraints and practical support for childcare.^42^ This highlights the importance of social support and childcare provision in creating opportunities for physical activity for mothers.^38^^,^^42^

We observed a progressive decrease in sitting time with higher parity, which is consistent with prior research.^43^ This may be related to demands of caring for young children, consistently with reports of increased household activities for parents with children.^38^ Although women who gave birth decreased sitting time more than those who remained nulliparous, they did not increase physical activity (Supplementary Table S7, available as Supplementary data at *IJE* online). This suggests that sedentary behaviour and physical activity are independent behaviours.^44^ Replacing sitting time with low-intensity activities can have significant health benefits, especially in less active individuals.^44^ In the current study, we observed an increase in weight in women who became parous despite decreases in sitting time. Conversely, Oken *et al.* reported positive associations of television viewing time with ≥5 kg post-partum weight retention^45^ whereas others reported no relationship between sitting time and post-partum weight retention or gain.^27^^,^^46^ One potential explanation for this discrepancy is that in our study we look at change in sitting time over a 6-year period, not just within the first post-partum year as did Oken *et al.* It is possible that any increase in energy expenditure from decreased sitting time in women who became parous was insufficient to make up for the positive energy balance resulting from decreased physical activity and increased energy intake.^47^

### Strengths and limitations

The strengths of this study include the large population-based sample, outcomes measured at two time points, longitudinal study design with sufficient follow-up time to allow multiple births and having the comparison group from the same birth cohort. Limitations include attrition over time in the ALSWH. The women included in the current study are less representative of the general population than when the cohort began, as women having less education, not born in Australia, with poorer health or who smoked were most commonly lost to follow-up. There may have been a selective loss of those with poorer health outcomes, leading to underestimation of the strength of associations. Women who gave birth during the study period may have reported lifestyle behaviours differently from those who did not give birth due to information on healthy lifestyles they may have received as part of antenatal care. This could potentially exaggerate the improvements in diet quality and sitting time, and underestimate how much energy intake and physical activity had worsened in parous relative to nulliparous women. Furthermore, all measures were self-reported, which could introduce some bias. However, a validation study from the 1946–1951 ALSWH cohort demonstrated substantial agreement between self-reported and measured weight and height.^48^ The other measures we used were also validated and shown to have acceptable psychometric properties.^21^^,^^23^^,^^49^^,^^50^

## Conclusion

This study provides insight into patterns of change in lifestyle behaviours in a national population cohort of women aged 31–36 years according to whether or not they gave birth over a 6-year period. Women who became parous increased weight and energy intake and decreased physical activity, but improved diet quality and sitting time, to a greater extent than those who remained nulliparous. The improvement in diet quality was only seen in women who became primiparous. These findings will inform future research on targeting improvements in weight, energy intake and physical activity for enhancing women’s health during the childbearing years and beyond.

## Ethics approval

Ethics approvals were granted by the University of Newcastle (H–076–0795) and the University of Queensland (200400224), and informed consent was received from all participants.

## Supplementary Material

dyac139_Supplementary_Data

## Data Availability

ALSWH survey data are owned by the Australian Government Department of Health and due to the personal nature of the data collected, release by ALSWH is subject to strict contractual and ethical restrictions. Ethical review of ALSWH is by the Human Research Ethics Committees at the University of Queensland and the University of Newcastle. De-identified data are available to collaborating researchers where a formal request to make use of the material has been approved by the ALSWH Data Access Committee. The committee is receptive of requests for data sets required to replicate results. Information on applying for ALSWH data is available from https://alswh.org.au/for-data-users/applying-for-data/.
