# Supplementary material for: Assessing patterns of change in lifestyle behaviours by parity: a longitudinal cohort study
Source: Int J Epidemiol. 2022 Jul 1;52(2):589–99. doi: 10.1093/ije/dyac139 (PMC10114083; doi:10.1093/ije/dyac139)
Supplement: dyac139_Supplementary_Data [file dyac139_supplementary_data.docx]

# **Supplement to: Assessing patterns of change in lifestyle behaviours by parity: a longitudinal cohort study**

Maureen Makama, Arul Earnest, Siew Lim, Helen Skouteris, Briony Hill, Helena Teede, Jacqueline Boyle, Wendy J. Brown, Allison M. Hodge, Lisa J. Moran

Supplementary Table S1. Components of the Dietary Guideline Index^1^

| Components/ Indicators | Dietary guideline | Description | Criteria for maximum score (10)^a^ | Criteria for minimum score (0)^a^ |
| --- | --- | --- | --- | --- |
| Variety | Enjoy a wide variety of nutritious foods | Dietary variety – proportion of foods for each core food group consumed at least once per week | 100% | 0 |
| Vegetables | Eat plenty of vegetables and legumes | Servings of vegetables and legumes per day | ≥5 | 0 |
| Fruit | Eat plenty of fruits | Servings of fruit per day | ≥2 | 0 |
| Cereals | Eat plenty of cereals (including breads, rice, pasta and noodles) | Frequency of consumption of breads and cereals per day | ≥4 | 0 |
| Whole grains | Eat preferable whole-grain cereals | Proportion of wholemeal or whole grain bread consumed relative to total bread | 100% | 0% |
| Protein | Include lean meat, fish, poultry, and/or alternatives | Frequency of consumption of meat and alternatives per day | ≥1 | 0 |
| Lean protein | Include lean meat, fish, poultry, and/or alternatives | Proportion of lean meats and alternatives relative to total meats and alternatives | 100% | 0% |
| Dairy | Include milks, yoghurts, cheeses and/or alternatives | Frequency of consumption of dairy products per day | ≥2 | 0 |
| Low-fat dairy | Reduced-fat varieties should be chosen where possible | Type of milk usually consumed | Low-fat milk | Whole milk |
| Saturated fat | Limit saturated fat and moderate total fat intake | Type of milk usually consumed + Trimming of fat from meat | Low-fat milk/Usually | Whole milk/Never or rarely |
| Alcohol | Limit alcohol intake | Frequency of consumption of all alcoholic beverages per day | 0 | ≥1 |
| Added sugars | Consume only moderate amounts of sugars and foods containing added sugars | Frequency of consumption of soft drink, cordial, fruit juice drink, jam, chocolate, confectionary per day | ≤1.25 | >1.25 |
| Extras (discretionary foods) | Eat according to your energy needs | Frequency of consumption of extra foods (not essential to provide nutrient requirements e.g. pastries and chips) per day | ≤2.5 | >2.5 |

^a^Serving unless otherwise indicated. Participants with intakes between the maximum and minimum were assigned scores proportionately

Supplementary Table S2. Participant characteristics at baseline (survey 3) grouped by parity status at survey 5

|  | Nulliparous at survey 5  (n = 2424) | Primiparous at survey 5  (n = 1090) | Multiparous at survey 5  (n = 1413) | *P-value* |
| --- | --- | --- | --- | --- |
| Age (years), Mean (SD) | 27.3 (1.4) | 27.4 (1.4) | 27.6 (1.4) | <0.001* |
| Weight (kg), Mean (SD) | 67.7 (16.2) | 65.2 (13.0) | 66.2 (12.7) | <0.001* |
| Height (cm), Mean (SD) | 165.7 (7.1) | 165.9 (7.3) | 166.3 (6.8) | 0.052 |
| BMI (kg/m^2^), Mean (SD) | 24.7 (5.7) | 23.7 (4.5) | 23.9 (4.4) | <0.001* |
| Country of birth |  |  |  | 0.226 |
| Australian born | 2225 (91.8) | 1015 (93.1) | 1315 (93.1) |  |
| Non-Australian born | 199 (8.2) | 75 (6.9) | 98 (6.9) |  |
| Marital status n (%) |  |  |  | <0.001 |
| Married/de facto | 731 (30.3) | 642 (59.1) | 1188 (84.3) |  |
| Separated/divorced/widowed | 53 (2.2) | 34 (3.1) | 20 (1.4) |  |
| Never married | 1629 (67.5) | 411 (37.8) | 202 (14.3) |  |
| Current smoker n (%) |  |  |  | <0.001 |
| No | 1797 (74.4) | 838 (77.1) | 1182 (83.9) |  |
| Yes | 617 (25.6) | 249 (22.9) | 227 (16.1) |  |
| Alcohol intake n (%) |  |  |  | 0.012 |
| Low risk drinker | 1648 (68.3) | 785 (72.2) | 978 (69.5) |  |
| Non-drinker | 149 (6.2) | 57 (5.2) | 75 (5.3) |  |
| Rarely drinks | 496 (20.6) | 204 (18.8) | 313 (22.2) |  |
| Risky/high risk drinker | 121 (5.0) | 41 (3.8) | 42 (3.0) |  |
| Education n (%) |  |  |  | 0.639 |
| No formal/high school | 475 (19.8) | 205 (19.2) | 250 (18.1) |  |
| Trade/diploma | 546 (22.8) | 257 (24.1) | 336 (24.3) |  |
| Degree or higher | 1377 (57.4) | 606 (56.7) | 798 (57.7) |  |
| Annual household income n (%) |  |  |  | <0.001 |
| No income | 4 (0.2) | 6 (0.7) | 3 (0.2) |  |
| Low | 296 (18.0) | 110 (12.5) | 103 (8.1) |  |
| Medium | 714 (43.3) | 354 (40.3) | 478 (37.6) |  |
| High | 635 (38.5) | 409 (46.5) | 688 (54.1) |  |
| Occupation n (%) |  |  |  | <0.001 |
| No paid job | 174 (7.3) | 49 (4.5) | 56 (4.0) |  |
| Clerical job | 473 (19.8) | 207 (19.1) | 235 (16.7) |  |
| Associate professional | 639 (26.7) | 273 (25.2) | 370 (26.4) |  |
| Professional | 1109 (46.3) | 553 (51.1) | 743 (52.9) |  |
| Stress level, Mean (SD) | 1.0 (0.6) | 0.9 (0.5) | 0.8 (0.4) | <0.001* |
| Depression n (%) |  |  |  | <0.001 |
| No | 1732 (72.4) | 842 (78.1) | 1157 (83.2) |  |
| Yes | 662 (27.7) | 236 (21.9) | 233 (16.8) |  |
| Anxiety n (%) |  |  |  | <0.001 |
| No | 1082 (44.9) | 493 (45.3) | 731 (52.0) |  |
| Yes | 1330 (55.1) | 595 (54.7) | 675 (48.0) |  |
| Energy intake (kJ/day), Mean (SD) | 6847.8 (2245.1) | 6808.1 (2262.2) | 6881.0 (2135.4) | 0.7199* |
| Total diet quality (units), Mean (SD) | 83.9 (12.0) | 84.4 (11.5) | 85.2 (10.9) | 0.0029* |
| Physical activity (MET.min/week), Mean (SD) | 1142.3 (1058.0) | 1069.0 (971.1) | 1004.1 (944.9) | <0.001* |
| Sitting time (hours/day), Mean (SD) | 6.9 (2.8) | 6.6 (2.8) | 6.5 (2.6) | <0.001* |
| ARIA+^a^ |  |  |  | 0.169 |
| Major cities of Australia | 1530 (63.4) | 685 (62.8) | 835 (59.3) |  |
| Inner regional Australia | 554 (22.9) | 247 (22.7) | 361 (25.6) |  |
| Outer regional Australia | 286 (11.8) | 128 (11.8) | 171 (12.1) |  |
| Remote Australia | 35 (1.5) | 23 (2.1) | 35 (2.5) |  |
| Very remote Australia | 10 (0.4) | 6 (0.6) | 7 (0.5) |  |

Data were analysed by one-way analysis of variance* for continuous variables and Chi-square or Fisher’s exact test for categorical variables as appropriate; SD – standard deviation; ^a^ARIA+ is Accessibility/Remoteness Index of Australia; BMI – Body Mass Index; MET – Metabolic Equivalent of Task.

Supplementary Table S3. Differences in macronutrients intake over follow-up time grouped by parity status at survey 5

| Parameter | Survey 3 Mean (SD) | Survey 5 Mean (SD) | ^a^Mean difference over time (95% CI) | *P*-value |
| --- | --- | --- | --- | --- |
| **Carbohydrate** (% energy) |  |  |  |  |
| Nulliparous at survey 5 | 41.2 (6.5) | 38.9 (6.5) | -2.3 (-2.6, -2.0) | <0.001 |
| Parous at survey 5 | 41.3 (6.2) | 39.8 (5.2) | -1.6 (-1.8, -1.3) | <0.001 |
| ^b^Unadjusted difference (Parous – Nulliparous) (n=4620) |  |  | 0.8 (0.5, 1.1) | <0.001 |
| ^c^Adjusted difference (Parous – Nulliparous) (n=4123) |  |  | 1.0 (0.7, 1.3) | <0.001 |
| **Protein** (% energy) |  |  |  |  |
| Nulliparous at survey 5 | 19.1 (3.2) | 20.0 (3.2) | 1.0 (0.8, 1.01) | <0.001 |
| Parous at survey 5 | 19.5 (3.1) | 19.9 (2.8) | 0.4 (0.3, 0.6) | <0.001 |
| ^b^Unadjusted difference (Parous – Nulliparous) (n=4620) |  |  | -0.3 (-0.5, -0.2) | <0.001 |
| ^c^Adjusted difference (Parous – Nulliparous) (n=4123) |  |  | -0.17 (-0.36, 0.01) | 0.060 |
| **Fat** (% energy) |  |  |  |  |
| Nulliparous at survey 5 | 32.6 (5.9) | 34.0 (5.4) | 1.4 (1.1, 1.6) | <0.001 |
| Parous at survey 5 | 32.5 (5.9) | 35.9 (4.6) | 3.4 (3.2, 3.6) | <0.001 |
| ^b^Unadjusted difference (Parous – Nulliparous) (n=4620) |  |  | 1.9 (1.7, 2.2) | <0.001 |
| ^c^Adjusted difference (Parous – Nulliparous) (n=4123) |  |  | 2.1 (1.8, 2.4) | <0.001 |
| **Saturated fat** (% energy) |  |  |  |  |
| Nulliparous at survey 5 | 13.3 (3.4) | 13.9 (3.1) | 0.6 (0.4, 0.7) | <0.001 |
| Parous at survey 5 | 13.4 (3.3) | 15.3 (2.9) | 1.9 (1.8, 2.1) | <0.001 |
| ^b^Unadjusted difference (Parous – Nulliparous) (n=4620) |  |  | 1.4 (1.3, 1.6) | <0.001 |
| ^c^Adjusted difference (Parous – Nulliparous) (n=4123) |  |  | 1.5 (1.3, 1.7) | <0.001 |
| **Monounsaturated fat** (% energy) |  |  |  |  |
| Nulliparous at survey 5 | 11.4 (2.3) | 12.2 (2.2) | 0.7 (0.6, 0.8) | <0.001 |
| Parous at survey 5 | 11.4 (2.3) | 12.6 (1.8) | 1.2 (1.1, 1.3) | <0.001 |
| ^b^Unadjusted difference (Parous – Nulliparous) (n=4620) |  |  | 0.5 (0.3, 0.6) | <0.001 |
| ^c^Adjusted difference (Parous – Nulliparous) (n=4123) |  |  | 0.5 (0.4, 0.6) | <0.001 |
| **Polyunsaturated fat** (% energy) |  |  |  |  |
| Nulliparous at survey 5 | 4.8 (1.8) | 4.9 (1.6) | 0.07 (-0.01, 0.15) | 0.0874 |
| Parous at survey 5 | 4.7 (1.7) | 4.9 (1.5) | 0.15 (0.07, 0.23) | <0.001 |
| ^b^Unadjusted difference (Parous – Nulliparous) (n=4620) |  |  | -0.02 (-0.10, 0.07) | 0.679 |
| ^c^Adjusted difference (Parous – Nulliparous) (n=4123) |  |  | -0.002 (-0.100, 0.110) | 0.976 |
| **Fibre** (g/day) |  |  |  |  |
| Nulliparous at survey 5 | 18.4 (7.9) | 18.6 (7.2) | 0.2 (-0.1, 0.5) | 0.1970 |
| Parous at survey 5 | 18.4 (7.2) | 20.6 (7.3) | 2.2 (1.9, 2.5) | <0.001 |
| ^b^Unadjusted difference (Parous – Nulliparous) (n=4746) |  |  | 2.0 (1.6, 2.4) | <0.001 |
| ^c^Adjusted difference (Parous – Nulliparous) (n=4230) |  |  | 2.2 (1.8, 2.7) | <0.001 |
| **Cholesterol** (mg/day) |  |  |  |  |
| Nulliparous at survey 5 | 234.5 (116.8) | 251.1 (116.3) | 16.6 (11.7, 21.5) | <0.001 |
| Parous at survey 5 | 236.1 (109.9) | 275.5 (106.7) | 39.4 (35.0, 43.9) | <0.001 |
| ^b^Unadjusted difference (Parous – Nulliparous) (n=4746) |  |  | 23.7 (18.1, 29.3) | <0.001 |
| ^c^Adjusted difference (Parous – Nulliparous) (n=4230) |  |  | 26.6 (20.1, 33.0) | <0.001 |
| **Alcohol** (g/day) |  |  |  |  |
| Nulliparous at survey 5 | 12.1 (14.2) | 12.0 (14.4) | -0.1 (-0.6, 0.5) | 0.8013 |
| Parous at survey 5 | 11.0 (12.5) | 6.6 (10.7) | -4.3 (-4.9, -3.9) | <0.001 |
| ^b^Unadjusted difference (Parous – Nulliparous) (n=4746) |  |  | -4.9 (-5.5, -4.3) | <0.001 |
| ^c^Adjusted difference (Parous – Nulliparous) (4240) |  |  | -5.9 (-6.6, -5.1) | <0.001 |
| **Glycaemic index** |  |  |  |  |
| Nulliparous at survey 5 | 52.3 (4.0) | 49.9 (3.8) | -2.4 (-2.6, -2.3) | <0.001 |
| Parous at survey 5 | 52.3 (3.9) | 51.1 (3.7) | -1.2 (-1.4, -1.0) | <0.001 |
| ^b^Unadjusted difference (Parous – Nulliparous) (n=4746) |  |  | 1.2 (1.0, 1.4) | <0.001 |
| ^c^Adjusted difference (Parous – Nulliparous) (n=4230) |  |  | 1.3 (1.1, 1.5) | <0.001 |
| **Glycaemic load** |  |  |  |  |
| Nulliparous at survey 5 | 92.6 (38.7) | 80.6 (32.7) | -12.0 (-13.6, -10.5) | <0.001 |
| Parous at survey 5 | 92.4 (37.0) | 95.5 (35.7) | 3.1 (1.5, 4.7) | <0.001 |
| ^b^Unadjusted difference (Parous – Nulliparous) (n=4746) |  |  | 15.0 (13.2, 16.8) | <0.001 |
| ^c^Adjusted difference (Parous – Nulliparous) (n=4230) |  |  | 16.4 (14.4, 18.5) | <0.001 |

CI – Confidence Interval; SD – Standard Deviation; ^a^Data analysed using paired *t*-test for the within group change; analysis of covariance was used to quantify the difference over time between nulliparous and parous women; ^b^Adjusted for the outcome measures at baseline; ^c^Additionally adjusted for age, BMI, marital status, education level, occupation category, smoking status, alcohol intake, mean stress level, depression and anxiety at baseline

Supplementary Table S4. Differences in micronutrients intake over follow-up time grouped by parity status at survey 5

| Parameter | Survey 3 Mean (SD) | Survey 5 Mean (SD) | ^a^Mean difference over time (95% CI) | *P*-value |
| --- | --- | --- | --- | --- |
| **Calcium** (mg/day) |  |  |  |  |
| Nulliparous at survey 5 | 829.9 (302.9) | 825.0 (285.5) | -4.9 (-17.2, 7.5) | 0.4386 |
| Parous at survey 5 | 849.0 (290.4) | 921.1 (297.8) | 72.1 (59.1, 85.1) | <0.001 |
| ^b^Unadjusted difference (Parous – Nulliparous) (n=4746) |  |  | 88.0 (73.0, 103.0) | <0.001 |
| ^c^Adjusted difference (Parous – Nulliparous) (n=4230) |  |  | 101.5 (84.2, 118.8) | <0.001 |
| **Iron** (mg/day) |  |  |  |  |
| Nulliparous at survey 5 | 11.1 (5.1) | 11.3 (4.6) | 0.25 (0.03, 0.47) | 0.0229 |
| Parous at survey 5 | 11.2 (4.7) | 12.7 (4.8) | 1.6 (1.4, 1.8) | <0.001 |
| ^b^Unadjusted difference (Parous – Nulliparous) (n=4746) |  |  | 1.4 (1.2, 1.6) | <0.001 |
| ^c^Adjusted difference (Parous – Nulliparous) (n=4230) |  |  | 1.6 (1.3, 1.9) | <0.001 |
| **Folate** (µg/day) |  |  |  |  |
| Nulliparous at survey 5 | 247.1 (101.4) | 240.7 (89.6) | -6.4 (-10.6, -2.2) | 0.0026 |
| Parous at survey 5 | 248.8 (9734) | 263.8 (93.2) | 15.1 (10.9, 19.3) | <0.001 |
| ^b^Unadjusted difference (Parous – Nulliparous) (n=4746) |  |  | 22.5 (17.8, 27.3) | <0.001 |
| ^c^Adjusted difference (Parous – Nulliparous) (n=4230) |  |  | 26.9 (21.4, 32.3) | <0.001 |
| **Sodium** (mg/day) |  |  |  |  |
| Nulliparous at survey 5 | 2199.9 (913.3) | 2090.1 (860.8) | -109.7 (-146.3, -73.1) | <0.001 |
| Parous at survey 5 | 2196.9 (861.5) | 2407.9 (837.6) | 211.7 (175.4, 248.1) | <0.001 |
| ^b^Unadjusted difference (Parous – Nulliparous) (n=4746) |  |  | 319.4 (276.3, 362.4) | <0.001 |
| ^c^Adjusted difference (Parous – Nulliparous) (n=4230) |  |  | 349.7 (300.3, 399.1) | <0.001 |
| **Zinc** (mg/day) |  |  |  |  |
| Nulliparous at survey 5 | 9.8 (4.4) | 10.1 (4.2) | 0.3 (0.1, 0.5) | 0.0013 |
| Parous at survey 5 | 10.1 (4.1) | 11.5 (4.0) | 1.4 (1.2, 1.6) | <0.001 |
| ^b^Unadjusted difference (Parous – Nulliparous) (n=4746) |  |  | 1.3 (1.0, 1.5) | <0.001 |
| ^c^Adjusted difference (Parous – Nulliparous) (n=4230) |  |  | 1.4 (1.1, 1.6) | <0.001 |
| **Magnesium** (mg/day) |  |  |  |  |
| Nulliparous at survey 5 | 259.3 (95.2) | 263.3 (89.8) | 4.0 (0.2, 7.9) | 0.0411 |
| Parous at survey 5 | 255.3 (88.5) | 286.3 (92.4) | 31.0 (27.1, 34.9) | <0.001 |
| ^b^Unadjusted difference (Parous – Nulliparous) (n=4746) |  |  | 24.7 (20.1, 29.4) | <0.001 |
| ^c^Adjusted difference (Parous – Nulliparous) (n=4230) |  |  | 29.0 (23.6, 34.3) | <0.001 |
| **Phosphorus** (mg/day) |  |  |  |  |
| Nulliparous at survey 5 | 1353.2 (496.3) | 1374.4 (472.3) | 21.3 (0.9, 41.6) | 0.0407 |
| Parous at survey 5 | 1363.0 (471.7) | 1519.2 (473.4) | 156.2 (136.0, 176.3) | <0.001 |
| ^b^Unadjusted difference (Parous – Nulliparous) (n=4746) |  |  | 140.4 (116.4, 164.5) | <0.001 |
| ^c^Adjusted difference (Parous – Nulliparous) (n=4230) |  |  | 159.9 (132.3, 187.6) | <0.001 |
| **Potassium** (mg/day) |  |  |  |  |
| Nulliparous at survey 5 | 2544.3 (865.4) | 2527.5 (818.8) | -16.9 (-52.3, 18.6) | 0.3504 |
| Parous at survey 5 | 2583.03 (838.7) | 2750.6 (811.8) | 167.6 (133.0, 202.1) | <0.001 |
| ^b^Unadjusted difference (Parous – Nulliparous) (n=4746) |  |  | 206.2 (164.9, 247.5) | <0.001 |
| ^c^Adjusted difference (Parous – Nulliparous) (n=4230) |  |  | 247.2 (199.7, 294.8) | <0.001 |
| **Beta-carotene** (µg/day) |  |  |  |  |
| Nulliparous at survey 5 | 3297.6 (1916.5) | 3427.9 (1869.5) | 130.3 (49.8, 210.7) | 0.0015 |
| Parous at survey 5 | 3372.1 (1855.4) | 3826.7 (1828.0) | 454.6 (376.5, 532.6) | <0.001 |
| ^b^Unadjusted difference (Parous – Nulliparous) (n=4746) |  |  | 366.3 (272.0, 460.6) | <0.001 |
| ^c^Adjusted difference (Parous – Nulliparous) (n=4230) |  |  | 388.6 (278.2, 499.0) | <0.001 |
| **Niacin** (mg/day) |  |  |  |  |
| Nulliparous at survey 5 | 19.3 (8.9) | 18.7 (8.2) | -0.6 (-1.0, -0.2) | 0.0022 |
| Parous at survey 5 | 19.5 (8.5) | 21.1 (8.2) | 1.7 (1.3, 2.0) | <0.001 |
| ^b^Unadjusted difference (Parous – Nulliparous) (n=4746) |  |  | 2.4 (1.9, 2.8) | <0.001 |
| ^c^Adjusted difference (Parous – Nulliparous) (n=4230) |  |  | 2.7 (2.2, 3.2) | <0.001 |
| **Retinol** (µg/day) |  |  |  |  |
| Nulliparous at survey 5 | 287.8 (157.3) | 271.2 (135.4) | -16.6 (-22.6, -10.6) | <0.001 |
| Parous at survey 5 | 286.7 (151.9) | 347.2 (149.6) | 60.6 (54.0, 67.1) | <0.001 |
| ^b^Unadjusted difference (Parous – Nulliparous) (n=4746) |  |  | 76.5 (69.2, 83.7) | <0.001 |
| ^c^Adjusted difference (Parous – Nulliparous) (n=4230) |  |  | 82.8 (74.4, 91.2) | <0.001 |
| **Riboflavin** (mg/day) |  |  |  |  |
| Nulliparous at survey 5 | 2.1 (0.9) | 2.1 (0.9) | -0.03 (-0.07, 0.01) | 0.1195 |
| Parous at survey 5 | 2.2 (0.9) | 2.5 (0.9) | 0.3 (0.2, 0.3) | <0.001 |
| ^b^Unadjusted difference (Parous – Nulliparous) (n=4746) |  |  | 0.3 (0.3, 0.4) | <0.001 |
| ^c^Adjusted difference (Parous – Nulliparous) (n=4230) |  |  | 0.4 (0.3, 0.4) | <0.001 |
| **Thiamine** (mg/day) |  |  |  |  |
| Nulliparous at survey 5 | 1.3 (0.6) | 1.3 (0.6) | -0.08 (-0.11, -0.06) | <0.001 |
| Parous at survey 5 | 1.4 (0.6) | 1.5 (0.6) | 0.2 (0.1, 0.2) | <0.001 |
| ^b^Unadjusted difference (Parous – Nulliparous) (n=4746) |  |  | 0.3 (0.2, 0.3) | <0.001 |
| ^c^Adjusted difference (Parous – Nulliparous) (n=4230) |  |  | 0.3 (0.2, 0.3) | <0.001 |
| **Vitamin C** (mg/day) |  |  |  |  |
| Nulliparous at survey 5 | 124.0 (71.4) | 106.0 (59.4) | -18.0 (-21.1, -15.0) | <0.001 |
| Parous at survey 5 | 127.7 (74.0) | 111.6 (60.4) | -16.1 (-19.2, -13.1) | <0.001 |
| ^b^Unadjusted difference (Parous – Nulliparous) (n=4746) |  |  | 4.5 (1.3, 7.7) | 0.005 |
| ^c^Adjusted difference (Parous – Nulliparous) (n=4230) |  |  | 7.1 (3.4, 10.8) | <0.001 |
| **Vitamin E** (mg/day) |  |  |  |  |
| Nulliparous at survey 5 | 5.4 (2.4) | 5.5 (2.2) | 0.15 (0.05, 0.25) | 0.0025 |
| Parous at survey 5 | 5.2 (2.1) | 6.0 (2.2) | 0.7 (0.7, 0.8) | <0.001 |
| ^b^Unadjusted difference (Parous – Nulliparous) (n=4746) |  |  | 0.5 (0.4, 0.6) | <0.001 |
| ^c^Adjusted difference (Parous – Nulliparous) (n=4230) |  |  | 0.6 (0.4, 0.7) | <0.001 |

CI – Confidence Interval; SD – Standard Deviation; ^a^Data analysed using paired *t*-test for the within group change; analysis of covariance was used to quantify the difference over time between nulliparous and parous women; ^b^Adjusted for the outcome measures at baseline; ^c^Additionally adjusted for age, BMI, marital status, education level, occupation category, smoking status, alcohol intake, mean stress level, depression and anxiety at baseline

Supplementary Table S5. Differences in diet quality components over follow-up time grouped by parity status at survey 5

| Parameter | Survey 3 Mean (SD) | Survey 5 Mean (SD) | ^a^Mean difference over time (95% CI) | *P*-value |
| --- | --- | --- | --- | --- |
| **DGI variety^d^** |  |  |  |  |
| Nulliparous at survey 5 | 4.8 (1.3) | 5.1 (1.3) | 0.4 (0.3, 0.4) | <0.001 |
| Parous at survey 5 | 4.9 (1.3) | 5.5 (1.3) | 0.6 (0.6, 0.7) | <0.001 |
| ^b^Unadjusted difference (Parous – Nulliparous) (n=4926) |  |  | 0.3 (0.3, 0.4) | <0.001 |
| ^c^Adjusted difference (Parous – Nulliparous) (n=4362) |  |  | 0.4 (0.3, 0.4) | <0.001 |
| **DGI vegetables** |  |  |  |  |
| Nulliparous at survey 5 | 3.9 (0.04) | 4.1 (0.04) | 0.2 (0.1, 0.3) | <0.001 |
| Parous at survey 5 | 3.9 (1.9) | 4.4 (1.9) | 0.4 (0.4, 0.5) | <0.001 |
| ^b^Unadjusted difference (Parous – Nulliparous) (n=4927) |  |  | 0.3 (0.2, 0.3) | <0.001 |
| ^c^Adjusted difference (Parous – Nulliparous) (n=4362) |  |  | 0.3 (0.2, 0.5) | <0.001 |
| **DGI fruit** |  |  |  |  |
| Nulliparous at survey 5 | 9.9 (0.93) | 9.9 (0.86) | 0.02 (-0.02, 0.06) | 0.4345 |
| Parous at survey 5 | 9.9 (0.86) | 10.0 (0.60) | 0.04 (0.01, 0.07) | 0.0165 |
| ^b^Unadjusted difference (Parous – Nulliparous) (n=4927) |  |  | 0.03 (-0.01, 0.07) | 0.090 |
| ^c^Adjusted difference (Parous – Nulliparous) (n=4362) |  |  | 0.050 (0.001, 0.090) | 0.045 |
| **DGI cereals** |  |  |  |  |
| Nulliparous at survey 5 | 5.4 (2.4) | 4.8 (2.3) | -0.6 (-0.7, -0.5) | <0.001 |
| Parous at survey 5 | 5.4 (2.3) | 5.7 (2.3) | 0.3 (0.2, 0.4) | <0.001 |
| ^b^Unadjusted difference (Parous – Nulliparous) (n=4927) |  |  | 0.9 (0.8, 1.0) | <0.001 |
| ^c^Adjusted difference (Parous – Nulliparous) (n=4362) |  |  | 1.0 (0.9, 1.1) | <0.001 |
| **DGI whole grains** |  |  |  |  |
| Nulliparous at survey 5 | 5.6 (4.9) | 7.9 (4.0) | 2.4 (2.1, 2.6) | <0.001 |
| Parous at survey 5 | 5.5 (5.0) | 7.7 (4.2) | 2.2 (2.0, 2.4) | <0.001 |
| ^b^Unadjusted difference (Parous – Nulliparous) (n=4927) |  |  | -0.26 (-0.48, -0.03) | 0.024 |
| ^c^Adjusted difference (Parous – Nulliparous) (n=4362) |  |  | -0.30 (-0.55, -0.04) | 0.015 |
| **DGI protein** |  |  |  |  |
| Nulliparous at survey 5 | 8.9 (2.0) | 9.4 (1.6) | 0.5 (0.4, 0.5) | <0.001 |
| Parous at survey 5 | 9.1 (1.8) | 9.6 (1.2) | 0.5 (0.4, 0.6) | <0.001 |
| ^b^Unadjusted difference (Parous – Nulliparous) (n=4926) |  |  | 0.2 (0.1, 0.2) | <0.001 |
| ^c^Adjusted difference (Parous – Nulliparous) (n=4362) |  |  | 0.2 (0.1, 0.3) | <0.001 |
| **DGI lean protein** |  |  |  |  |
| Nulliparous at survey 5 | 8.5 (1.1) | 8.6 (0.99) | 0.13 (0.08, 0.17) | <0.001 |
| Parous at survey 5 | 8.5 (1.0) | 8.3 (0.99) | -0.1 (-0.2, -0.1) | <0.001 |
| ^b^Unadjusted difference (Parous – Nulliparous) (n=4921) |  |  | -0.3 (-0.3, -0.2) | <0.001 |
| ^c^Adjusted difference (Parous – Nulliparous) (n=4357) |  |  | -0.3 (-0.3, -0.2) | <0.001 |
| **DGI dairy** |  |  |  |  |
| Nulliparous at survey 5 | 7.4 (2.4) | 7.5 (2.3) | 0.14 (0.04, 0.24) | 0.0083 |
| Parous at survey 5 | 7.6 (2.2) | 8.1 (2.1) | 0.5 (0.4, 0.6) | <0.001 |
| ^b^Unadjusted difference (Parous – Nulliparous) (n=4927) |  |  | 0.5 (0.4, 0.6) | <0.001 |
| ^c^Adjusted difference (Parous – Nulliparous) (n=4362) |  |  | 0.6 (0.4, 0.7) | <0.001 |
| **DGI low fat dairy** |  |  |  |  |
| Nulliparous at survey 5 | 6.1 (4.0) | 6.2 (3.9) | 0.09 (-0.07, 0.26) | 0.2776 |
| Parous at survey 5 | 6.0 (3.9) | 4.7 (4.0) | -1.2 (-1.4, -1.9) | <0.001 |
| ^b^Unadjusted difference (Parous – Nulliparous) (n=4919) |  |  | -1.4 (-1.6, -1.2) | <0.001 |
| ^c^Adjusted difference (Parous – Nulliparous) (n=4357) |  |  | -1.5 (-1.7, -1.2) | <0.001 |
| **DGI saturated fat** |  |  |  |  |
| Nulliparous at survey 5 | 7.9 (1.3) | 8.1 (1.2) | 0.13 (0.08, 0.19) | <0.001 |
| Parous at survey 5 | 8.0 (1.2) | 8.2 (1.1) | 0.2 (0.1, 0.2) | <0.001 |
| ^b^Unadjusted difference (Parous – Nulliparous) (n=4921) |  |  | 0.10 (0.04, 0.16) | 0.002 |
| ^c^Adjusted difference (Parous – Nulliparous) (n=4357) |  |  | 0.15 (0.08, 0.22) | <0.001 |
| **DGI alcohol** |  |  |  |  |
| Nulliparous at survey 5 | 6.9 (4.3) | 6.8 (4.3) | -0.07 (-0.25, 0.10) | 0.4212 |
| Parous at survey 5 | 7.1 (4.2) | 8.5 (3.3) | 1.4 (1.2, 1.6) | <0.001 |
| ^b^Unadjusted difference (Parous – Nulliparous) (n=4927) |  |  | 1.6 (1.4, 1.8) | <0.001 |
| ^c^Adjusted difference (Parous – Nulliparous) (n=4372) |  |  | 1.9 (1.6, 2.1) | <0.001 |
| **DGI added sugars** |  |  |  |  |
| Nulliparous at survey 5 | 8.4 (3.7) | 8.4 (3.7) | 0.004 (-0.170, 0.180) | 0.9632 |
| Parous at survey 5 | 8.7 (3.4) | 7.8 (4.2) | -0.91 (-1.09, -0.73) | <0.001 |
| ^b^Unadjusted difference (Parous – Nulliparous) (n=4927) |  |  | -0.73 (-0.95, -0.52) | <0.001 |
| ^c^Adjusted difference (Parous – Nulliparous) (n=4362) |  |  | -0.79 (-1.03, -0.54) | <0.001 |
| **DGI extras (discretionary foods)** |  |  |  |  |
| Nulliparous at survey 5 | 0.28 (1.7) | 0.40 (2.0) | 0.11 (0.02, 0.20) | 0.0141 |
| Parous at survey 5 | 0.22 (1.5) | 0.16 (1.3) | -0.06 (-0.13, 0.01) | 0.070 |
| ^b^Unadjusted difference (Parous – Nulliparous) (n=4927) |  |  | -0.22 (-0.31, -0.13) | <0.001 |
| ^c^Adjusted difference (Parous – Nulliparous) (n=4362) |  |  | -0.17 (-0.27, -0.07) | 0.001 |

CI – Confidence Interval; SD – Standard Deviation; ^a^Data analysed using paired *t*-test for the within group change; analysis of was used to quantify the difference over time between nulliparous and parous women; ^b^Adjusted for the outcome measures at baseline; ^c^Additionally adjusted for age, BMI, marital status, education level, occupation category, smoking status, alcohol intake, mean stress level, depression, anxiety and index of accessibility/remoteness at baseline; ^d^Proportions of food for each core food group that are consumed at least once per week

Supplementary Table S6. Estimated marginal mean weight, energy intake, total diet quality, physical activity and sitting time in nulliparous, primiparous and multiparous women

|  | Nulliparous (Mean (95%CI)) | Primiparous (Mean (95%CI)) | Multiparous (Mean (95%CI)) |
| --- | --- | --- | --- |
| Marginal mean weight (kg) | 71.5 (70.8, 72.2) ^a^ | 72.6 (71.8, 73.4) ^b^ | 72.4 (71.6, 73.3) ^b^ |
| Marginal mean energy intake (kJ/day) | 6538.3 (6361.0, 6715.6) ^a^ | 7241.5 (7042.7, 7440.2) ^b^ | 7521.1 (7315.9, 7726.4) ^c^ |
| Marginal mean total diet quality | 85.1 (83.9, 86.4) ^a^ | 86.4 (86.0, 88.7) ^b^ | 85.7 (84.4, 87.1) ^a^ |
| Marginal mean physical activity (MET.min/week) | 932.1 (850.4, 1013.8) ^a^ | 536.7 (445.1, 628.3) ^b^ | 516.1 (421.6, 610.5) ^b^ |
| Marginal mean sitting time (hours/day) | 6.9 (6.6, 7.1) ^a^ | 5.6 (5.3, 5.8) ^b^ | 4.6 (4.3, 4.8) ^c^ |
| Marginal mean DGI variety^d^ | 5.0 (4.9, 5.1) ^a^ | 5.3 (5.2, 5.5) ^b^ | 5.3 (5.2, 5.5) ^b^ |
| Marginal mean DGI vegetables | 4.3 (4.1, 4.5) ^a^ | 4.5 (4.3, 4.7) ^b^ | 4.6 (4.4, 4.8) ^b^ |
| Marginal mean DGI fruit | 9.9 (9.8, 10.0) ^a^ | 9.9 (9.8, 10.0) ^a^ | 10.0 (9.9, 10.1) ^a^ |
| Marginal mean DGI cereal | 4.5 (4.2, 4.8) ^a^ | 5.5 (5.2, 5.7) ^b^ | 5.6 (5.3, 5.9) ^b^ |
| Marginal mean DGI whole grain | 6.8 (6.3, 7.3) ^a^ | 6.7 (6.1, 7.2) ^a^ | 6.3 (5.8, 6.9) ^b^ |
| Marginal mean DGI protein | 9.5 (9.3, 9.6) ^a^ | 9.7 (9.5, 9.8) ^b^ | 9.7 (9.5, 9.9) ^b^ |
| Marginal mean DGI lean protein | 8.5 (8.4, 8.7) ^a^ | 8.4 (8.3, 8.5) ^b^ | 8.1 (8.0, 8.3) ^c^ |
| Marginal mean DGI dairy | 7.2 (6.9, 7.5) ^a^ | 7.8 (7.6, 8.1) ^b^ | 7.7 (7.4, 8.0) ^b^ |
| Marginal mean DGI low fat dairy | 5.9 (5.4, 6.4) ^a^ | 4.7 (4.2, 5.2) ^b^ | 4.2 (3.7, 4.7) ^c^ |
| Marginal mean DGI saturated fat | 7.9 (7.7, 8.0) ^a^ | 8.1 (8.0, 8.3) ^b^ | 7.9 (7.8, 8.1) ^a^ |
| Marginal mean DGI alcohol | 6.4 (6.0, 6.9) ^a^ | 8.3 (7.8, 8.7) ^b^ | 8.3 (7.9, 8.8) ^b^ |
| Marginal mean DGI added sugars | 8.8 (8.3, 9.3) ^a^ | 8.3 (7.8, 8.9) ^b^ | 7.7 (7.1, 8.2) ^c^ |
| Marginal mean DGI extras (discretionary foods) | 0.4 (0.2, 0.6) ^a^ | 0.20 (-0.02, 0.42) ^b^ | 0.17 (-0.05, 0.40) ^b^ |

Data are reported as marginal means and 95% confidence intervals of the endpoints estimated by parity categories (nulliparous, primiparous and multiparous) and were analysed by analysis of covariance adjusted for endpoints at baseline (Survey 3), age, BMI, marital status, education level, occupation category, smoking status, alcohol intake, mean stress level, depression and anxiety at baseline (diet quality models also adjusted for ARIA+); Means in a row not sharing the same superscript letter differ at p<0.05 analysed using analysis of covariance and Tukey’s post hoc comparison test; DGI – Dietary Guideline Index; CI – confidence interval; MET – Metabolic Equivalent of Task; ^d^Proportions of food for each core food group that are consumed at least once per week;

Supplementary Table S7. Adjusted differences in weight, energy intake, diet quality, physical activity, sitting time and DGI components over follow-up time by parity status at survey 5

| Parameters | ^a^Mean difference over time (95% CI) | *P* value^a^ |
| --- | --- | --- |
| Weight (kg) (n=4351) |  |  |
| Nulliparous | ref |  |
| Primiparous | 1.1 (0.5, 1.7) | <0.001 |
| Multiparous | 0.9 (0.3, 1.6) | 0.004 |
| Energy intake (kJ/day) (n=4263) |  |  |
| Nulliparous | ref |  |
| Primiparous | 703.2 (553.7, 852.7) | <0.001 |
| Multiparous | 982.8 (827.3, 1138.4) | <0.001 |
| Physical activity (MET.min/week) (n=4222) |  |  |
| Nulliparous | ref |  |
| Primiparous | -395.4 (-465.0, -325.9) | <0.001 |
| Multiparous | -416.0 (-488.6, -343.5) | <0.001 |
| Sitting time (hours/day) (n=3959) |  |  |
| Nulliparous | ref |  |
| Primiparous | -1.3 (-1.5, -1.1) | <0.001 |
| Multiparous | -2.3 (-2.5, -2.1) | <0.001 |
| Total diet quality (n=4253) |  |  |
| Nulliparous | ref |  |
| Primiparous | 2.2 (1.5, 3.0) | <0.001 |
| Multiparous | 0.6 (-0.2, 1.4) | 0.137 |
| DGI variety^b^ (n=4362) |  |  |
| Nulliparous | ref |  |
| Primiparous | 0.3 (0.3, 0.4) | <0.001 |
| Multiparous | 0.3 (0.3, 0.4) | <0.001 |
| DGI vegetables (n=4362) |  |  |
| Nulliparous | ref |  |
| Primiparous | 0.21 (0.08, 0.33) | 0.001 |
| Multiparous | 0.3 (0.2, 0.4) | <0.001 |
| DGI fruit (n=4362) |  |  |
| Nulliparous | ref |  |
| Primiparous | 0.04 (-0.01, 0.09) | 0.140 |
| Multiparous | 0.052 (-0.003, 0.107) | 0.063 |
| DGI cereal (n=4362) |  |  |
| Nulliparous | ref |  |
| Primiparous | 0.9 (0.8, 1.1) | <0.001 |
| Multiparous | 1.0 (0.9, 1.2) | <0.001 |
| DGI whole grain (n=4362) |  |  |
| Nulliparous | ref |  |
| Primiparous | -0.1 (-0.4, 0.2) | 0.347 |
| Multiparous | -0.5 (-0.8, -0.2) | 0.003 |
| DGI protein (n=4362) |  |  |
| Nulliparous | ref |  |
| Primiparous | 0.2 (0.1, 0.3) | <0.001 |
| Multiparous | 0.2 (0.1, 0.3) | <0.001 |
| DGI lean protein (n=4357) |  |  |
| Nulliparous | ref |  |
| Primiparous | -0.16 (-0.23, -0.09) | <0.001 |
| Multiparous | -0.4 (-0.5, -0.3) | <0.001 |
| DGI dairy (n=4362) |  |  |
| Nulliparous | ref |  |
| Primiparous | 0.6 (0.5, 0.8) | <0.001 |
| Multiparous | 0.5 (0.3, 0.7) | <0.001 |
| DGI low fat dairy (n=4357) |  |  |
| Nulliparous | ref |  |
| Primiparous | -1.2 (-1.5, -1.0) | <0.001 |
| Multiparous | -1.7 (-2.0, -1.4) | <0.001 |
| DGI saturated fat (n=4357) |  |  |
| Nulliparous | ref |  |
| Primiparous | 0.2 (0.2, 0.3) | <0.001 |
| Multiparous | 0.05 (-0.04, 0.14) | 0.265 |
| DGI alcohol (n=4372) |  |  |
| Nulliparous | ref |  |
| Primiparous | 1.8 (1.6, 2.1) | <0.001 |
| Multiparous | 1.9 (1.6, 2.2) | <0.001 |
| DGI added sugars (n=4362) |  |  |
| Nulliparous | ref |  |
| Primiparous | -0.5 (-0.8, -0.2) | 0.001 |
| Multiparous | -1.1 (-1.4, -0.8) | <0.001 |
| DGI extras (discretionary foods) (n=4362) |  |  |
| Nulliparous | ref |  |
| Primiparous | -0.16 (-0.28, -0.03) | 0.012 |
| Multiparous | -0.18 (-0.31, -0.06) | 0.004 |

^a^Analysis of covariance model was used to quantify the changes over time for women who became primiparous or multiparous relative to those who remained nulliparous; ^b^Proportions of food for each core food group that are consumed at least once per week; Adjusted for age, BMI, marital status, education level, occupation category, smoking status, alcohol intake, mean stress level, depression and anxiety at baseline; Diet quality and components additionally adjusted for index of accessibility/remoteness; CI – confidence interval; MET – Metabolic Equivalent of Task.

Supplementary Table S8. Adjusted differences over follow-up time in parous vs nulliparous women in sensitivity analysis excluding women who were pregnant at survey 5

|  | ^a^Mean difference over time (95% CI) | *P* value |
| --- | --- | --- |
| **Weight** (kg) (n=3781) | 1.1 (0.6, 1.7) | <0.001 |
| **Energy intake** (kJ/day) (n=3706) | 915.0 (776.1, 1053.9) | <0.001 |
| **Total diet quality** (units) (n=3700) | 1.7 (1.0, 2.4) | <0.001 |
| **Physical activity** (MET.min/week) (n=3668) | -413.2 (-478.7, -347.7) | <0.001 |
| **Sitting time** (hours/day) (n=3442) | -1.7 (-1.9, -1.6) | <0.001 |

^a^Adjusted for age, body mass index, marital status, education level, occupation category, smoking status, alcohol intake, mean stress level, depression, anxiety, index of accessibility/remoteness and outcomes at baseline; CI – confidence Interval; SD – Standard Deviation; MET – Metabolic Equivalent of Task; Supplementary Table S9. Adjusted differences over follow-up time in parous vs nulliparous women in sensitivity analysis with imputed weight and BMI data

|  | Mean difference over time (95% CI) | P-value |
| --- | --- | --- |
| **Weight** (kg) (n=4638) | 1.0 (0.4, 1.5) | <0.001 |
| **Energy intake** (kJ/day) (n=4536) | 787.2 (661.5, 912.8) | <0.001 |
| **Total diet quality** (units) (n=4526) | 1.2 (0.6, 1.8) | <0.001 |
| **Physical activity** (MET.min/week) (n=4495) | -388.5 (-446.6, -330.5) | <0.001 |
| **Sitting time** (hours/day) (n=4232) | -1.8 (-1.9, -1.6) | <0.001 |

Multiple imputation of weight and BMI data by chained equations using a missing at random assumption; 20 imputations were performed; Imputation models included age, body mass index, marital status, education level, occupation category, smoking status, alcohol intake, mean stress level, depression and anxiety at baseline; Diet quality and components additionally included index of accessibility/remoteness; body mass index was not included in the model where weight was the outcome; CI – confidence interval; MET – Metabolic Equivalent of Task.

**References**

1. McNaughton SA, Ball K, Crawford D, Mishra GD. An index of diet and eating patterns is a valid measure of diet quality in an Australian population. *J Nutr*. 2008;138(1):86-93.
